# Associations Between Negative Body Image and Sexual Health Practices in Emerging Adults from Malaysia

**DOI:** 10.1007/s10508-024-02810-y

**Published:** 2024-02-06

**Authors:** Pei Hwa Goh, Tamara Luginbuehl, Viren Swami

**Affiliations:** 1 Department of Psychology, Jeffrey Cheah School of Medicine and Health Sciences, Monash University Malaysia, Jalan Lagoon Selatan, Bandar Sunway, 47500 Selangor Malaysia; 2 Department of Psychology, Stony Brook University, Stony Brook, NY USA; 3 School of Psychology and Sport Science, Anglia Ruskin University, Cambridge, UK; 4 Centre for Psychological Medicine, Perdana University, Kuala Lumpur, Malaysia

**Keywords:** Body dissatisfaction, Genital image, Risky sexual behavior, Condom use, HIV screening

## Abstract

Past findings on the association between negative body image and sexual health behaviors have been mixed and mostly derived from Western samples. The aim of the current study was to examine associations between indices of negative body image and sexual health practices that reduce the risk of disease transmission in emerging adults, and whether these associations are moderated by gender. Using an online survey, a convenience sample of 584 Malaysian adults aged 18–30 years (230 men, 354 women) completed measures of satisfaction with overall appearance, body size dissatisfaction, weight satisfaction, height satisfaction, and genital image evaluation. They also reported if they ever had partnered sex, condomless sex, and been screened for human immunodeficiency virus (HIV), as well as their lifetime number of penetrative sex partners. Hierarchical logistic and ordinal regression analyses indicated that more positive genital image evaluation, but not the other body image indices, was significantly associated with having had partnered sex and fewer lifetime penetrative sex partners. None of the body image indices were significantly associated with condomless sex. All associations were consistent across men and women. For HIV testing, a significant interaction between genital image evaluation and gender emerged. However, this was reduced to non-significance after controlling for the number of penetrative sex partners. Overall, our findings underline the importance of promoting improved genital image in interventions aimed at increasing positive sexual health behaviors.

**Supplementary Information:**

The online version contains supplementary material available at 10.1007/s10508-024-02810-y.

## Introduction

Reducing the transmission of sexually transmitted infections (STIs), including human immunodeficiency virus (HIV), is an important aspect of sexual health maintenance (World Health Organization, n.d.). This can be achieved through prevention-based practices, such as abstinence from sex, reducing one’s number of sexual partners, and using condoms during sex, as well as early detection through screening for HIV/STIs (Centers for Disease Control and Prevention, 2022). Such practices are particularly important in emerging adulthood (ages 18–29 years; Arnett et al., 2014), a key development period often characterized by the development, exploration, and integration of romantic and sexual relationships (Swami et al., 2022; Watkins & Beckmeyer, 2020). Indeed, some scholars have suggested that casual sexual relationships and experiences tend to increase in frequency during emerging adulthood (Claxton & van Dulmen, 2013), which could be viewed as risky sexual behavior because the expansion of one’s sexual network increases the risk of the spread of STIs if no protective measures are used (Rogers et al., 2002; Senn, 2013).

One factor that is known to play a role in shaping sexual health practices is body image, a multifaceted psychological experience of embodiment encompassing affective, cognitive, perceptual, and behavioral aspects related to one’s body and appearance (Cash & Smolak, 2011). In the current work, we focus on negative body image, which encompasses one’s dissatisfaction, excessive concern, or discontent with the body and appearance (Cash et al., 2004a, 2004b), and which is conceptually distinct from indices of positive body image (Tylka & Wood-Barcalow, 2015). Indeed, there is now a large body of evidence examining associations between negative body image and sexual health outcomes (for reviews, see Blashill & Safren, 2015; Woertman & van den Brink, 2012). Studies have reported significant associations between negative body image and risky sexual behaviors, including a lower probability of using contraceptives and earlier age of first intercourse (Eisenberg et al., 2005; Gillen et al., 2006). Additionally, negative body image is significantly associated with lower condom use self-efficacy (i.e., confidence in using condoms appropriately, as well as assertiveness and consistency in condom use during sex; Blashill & Safren, 2015).

There are several possible explanations for the significant association between negative body image and sexual health practices. Objectification theory (Fredrickson & Roberts, 1997) posits that one’s tendency to self-objectify or internalize observers’ perspectives of the physical self could increase negative feelings about one’s body (e.g., shame, anxiety) and a preoccupation with body monitoring. These objectifying thoughts and their associated negative emotions consume cognitive resources, which leave individuals with fewer attentional resources to attend to their sexual needs and bodily sensations (Ackard et al., 2000; Schooler et al., 2005). Additionally, the exposure of bodies during sex may increase the salience of concerns about one’s body and self-objectification tendencies among those with negative body image. In such contexts, negative feelings and thoughts about one’s body image may increase fear of negative appearance-based evaluation (Cash et al., 2004a, 2004b), hence promoting the engagement of maladaptive coping strategies to reduce the likelihood of being rejected by their sexual partner(s) (Blashill & Safren, 2015). Such strategies may manifest as reduced assertiveness concerning condom use, a lower likelihood of initiating conversations around safer sex, and the increased use of substances (e.g., alcohol) before or during sex (Blashill & Safren, 2015), which could lead to condomless sex. The depletion of attentional resources in those with negative body image in sexual situations may also leave fewer cognitive resources available to practice risk-reducing sexual behaviors (Parent & Moradi, 2015).

Aside from risk-reducing practices, timely or frequent screening for HIV/STIs is also an important practice for reducing the transmission of STIs and improving disease prognosis (May, 2017). Based on the principles of objectification theory (Fredrickson & Roberts, 1997), individuals who feel more negatively about their bodies might be expected to be more motivated to avoid confrontation with their source of distress (e.g., Swami & Furnham, 2018). This motivation to prevent the exposure of one’s body or information about one’s body to healthcare providers and oneself, in turn, reduces the likelihood of healthcare visits and HIV screening behaviors (Schick et al., 2010). Even though objectification theory was initially developed to understand the bodily experiences of women, there is evidence suggesting its application among men (Moradi & Huang, 2008). Within the context of sexual health, Ramseyer Winter et al. (2020) found that, among men who engage in casual sex, more negative body image was associated with a lower likelihood of ever having tested for HIV and other STIs. However, findings related to the application of objectification theory to men’s bodily experiences have been inconsistent (Davids et al., 2019; Gillen & Markey, 2019) and lacking.

Similarly, there is mixed support for the link between body image and sexual health in the literature (Gillen & Markey, 2019). While some studies have found that more negative body image (e.g., poorer genital image evaluation; DeMaria et al., 2011) is associated with a lower likelihood of sexual health screening in women, other studies have reported no significant association between indices of negative body image (e.g., body dissatisfaction; Gillen & Markey, 2014) and HIV screening behaviors in undergraduate men and women. The mixed support for the association between body image and sexual health outcomes may be due to the variability in body image indices used (Gillen & Markey, 2019), which range from satisfaction with overall appearance (Gillen et al., 2006), objectified body consciousness (i.e., concern about physical appearance, body shame; Hibbert et al., 2020) to body image self-consciousness (Ramseyer Winter et al., 2020). In addition, findings from studies that have examined multiple body image facets simultaneously have identified differential predictive capabilities across the facets. For instance, DeMaria et al. (2011) found that women’s gynecological screening behaviors were better predicted by dissatisfaction with their genital image, rather than general body dissatisfaction.

Additionally, studies have suggested varying associations between body image and sexual health behaviors among women and men. In women, indices of negative body image have been consistently linked with poor sexual health behaviors, including lower condom use self-efficacy and actual condom use (e.g., Corona et al., 2019; Gillen et al., 2006). In men, however, the evidence base is more equivocal. Some studies have found that men who evaluated their appearance more negatively were less likely to report engagement in condomless sex and reported fewer lifetime sex partners (Gillen et al., 2006), suggesting that negative body image may act as a protective factor rather than a risk factor for sexual health. On the other hand, some studies have found no association between indices of body image and condom use attitudes and self-efficacy in male emerging adults (e.g., Corona et al., 2019).

### Sexual Health Practices in Malaysia

Notably, the majority of studies on body image and sexual health practices have been conducted with White, relatively higher socioeconomic status (SES) participants from the United States, which calls into question the generalizability of extant findings (Henrich et al., 2010). Here, we focus on emerging adults in Malaysia, a multiethnic country in Southeast Asia, where STIs and unplanned pregnancies remain a public health concern. This is unsurprising given the taboos that surround the topic of sex and the lack of sexual health knowledge among Malaysians (Wong, 2012). For instance, Soleymani et al. (2015) surveyed 434 postgraduate students in Malaysia aged 20–46 years and found that, while 98% knew about AIDS, only 24% knew about chlamydia. Most participants believed that STIs were spread through handshakes (92%) and public toilets (77%). This is particularly worrying because the current sex education curriculum in Malaysia is not comprehensive and is also met with barriers that are deeply rooted in cultural and religious beliefs (Wong, 2012) despite the evidence that young Malaysians are engaging in risky sexual behaviors (National Health and Morbidity Survey, 2018) and despite parental demands for early sex education (Abdullah et al., 2020). Moreover, the Ministry of Health Malaysia (2022) has reported that the prevalence of HIV reported cases is the highest in adults aged 20–29 years.

Although research suggests that adults in Asian nations, including Malaysia, consider sex to be an important part of life (Tan et al., 2009), cultural norms often mean they are also less likely to be sexually experienced, satisfied with sexuality-related body image, and confident in practicing safe sex behaviors relative to adults in Western nations (Kaneko, 2007). In such contexts, it is possible that body image may have a weaker effect on sexual health given their generally poorer sexual health knowledge and practices—resulting in a floor effect. Examining associations between body image and sexual health practices in Malaysia, therefore, offers an opportunity to expand the previous focus on Western samples and to consider the cultural generalizability of extant findings.

### The Present Study

Given the combination of low sexual literacy (Soleymani et al., 2015) and high HIV rates in emerging adults in Malaysia, it is important to know more about the antecedents of sexual health practices that reduce HIV/STI transmission in this population. In the current work, therefore, we examined whether negative body image was associated with such practices in a sample of urban Malaysian emerging adults and whether these associations were moderated by gender. In terms of body image variables, we included measures of appearance satisfaction, body size dissatisfaction, satisfaction with weight, and evaluation of genital image. We also examined one’s satisfaction with height as height has been found to be an important source of body dissatisfaction in Asian communities (Mellor et al., 2013). This allowed us to comprehensively capture the multifaceted construct of negative body image *vis-à-vis* constructs that were most relevant to sexual health practices. In terms of sexual health practices, which were our outcome variables, we focused on one’s experience of partnered sex, condomless sex, number of lifetime sex partners, and HIV testing. Due to the relatively exploratory nature of the current work, we tested a general hypothesis that (H1) negative body image would be associated with poorer sexual health practices, such that lower satisfaction with overall appearance, weight, height, genital image, and body size would be associated with a higher likelihood of ever having partnered sex and condomless sex, a lower likelihood of ever testing for HIV, and more lifetime sex partners, and (H2) that gender would moderate these effects.

In our analyses, we also accounted for the role of potential confounding variables. First, because of the role of religiosity in sexual practices (Koletić et al., 2021; Lefkowitz et al., 2004) and the accessibility of sexual health knowledge (Wong, 2012), it was included in the current study as a control variable. People in exclusive relationships may engage more in condomless sex because they believe that STI risks are lower due to increased trust in their partner (East et al., 2007). We thus included past and current experiences with romantic relationships as a control variable. Other control variables were demographic variables that have been shown to be correlated with HIV testing, such as age and drug use (Hibbert et al., 2020).

## Method

### Participants

The initial participant pool consisted of 780 individuals who completed an online survey in English. However, after removing duplicates and responses that were more than 30% incomplete (*n* = 159) and/or inconsistent in their reporting of partnered sexual experience (*n* = 37), we were left with 584 participants (230 men, 354 women) who were 22.11 years old on average (*SD* = 2.43) and mostly identified as heterosexual (82.7%) and racially Chinese (59.2%) (see Table 1 for further demographic information). Of the 584 participants, 300 reported lifetime experience with oral and/or penetrative sex. This is within the sample size required to achieve a power of 0.80 based on various computations with G*Power 3.1 (Faul et al., 2009), which is between 223 following parameters based on Ramseyer Winter et al. (2020; *OR* = 0.98 with *M* = 21.98 and *SD* = 20.29 for their body image measure) and 382 based on Gillen et al. (2006; the increase of *f*^2^ = 0.03 obtained with the addition of interaction terms estimated for our five two-way interaction terms between body image and gender).

**Table 1:** Demographic characteristics of participants

Characteristic	Overall (*N* = 584)		With partnered sex experience (*N* = 300)	
	N	%	N	%
*Gender*				
Man	230	39.4	134	44.7
Woman	354	60.6	166	55.3
*Race*				
Malay	107	18.3	50	16.7
Chinese	346	59.2	174	58.0
Indian	90	15.4	53	17.7
Other	41	7.1	23	7.6
*Sexual orientation*				
Heterosexual	483	82.7	254	84.7
Bisexual	53	9.1	31	10.3
Homosexual	22	3.8	13	4.3
Asexual or graysexual	26	4.5	2	0.7
*Relationship status*				
Not in an exclusive relationship	366	62.7	122	40.7
In an exclusive relationship	218	37.3	178	59.3
*Relationship experience*				
Never been in a relationship	164	28.1	16	5.3
Have been in a relationship	420	71.9	284	94.7
*Religion*				
Islam	116	20.1	56	18.9
Buddhism/Taoism	201	34.9	99	33.4
Hinduism	48	8.3	26	8.8
Christian/Catholic	153	26.6	75	25.3
Agnostic/Atheist	58	10.1	40	13.5
Missing	8		4	
*How frequently do you use recreational drugs?*				
Never	536	91.8	260	86.7
Less than once a week	31	5.3	24	8.0
Once a week	6	1.0	5	1.7
Multiple times a week	4	0.7	4	1.3
Everyday	7	1.2	7	2.3

### Measures

#### Appearance Satisfaction

The appearance evaluation subscale of the multidimensional body-self relations questionnaire (MBSRQ; Cash, 2000) was used to measure one’s general satisfaction with their overall appearance. This subscale consists of seven items (e.g., “My body is sexually appealing”) rated on a scale of 1 (*strongly disagree*) to 5 (*strongly agree*)*.* Because the English version of the MBSRQ has not been validated with Malaysian samples (but see Swami et al., 2019), an exploratory factor analysis (EFA) was run to examine the scale’s factorial validity separately for men and women (see Table S1). Based on the finding that a single factor should be extracted, we computed an overall appearance satisfaction score as the mean of all seven items, where higher scores indicated more positive evaluations of overall appearance, ω = 0.88 (95% CI = 0.87, 0.90).

#### Genital Image Evaluation

The abbreviated 4-item version of the female genital self-image scale (FGSIS-4; Herbenick et al., 2011) was used to assess participants’ feelings and beliefs about their genitals. Participants indicated the extent to which each of the four statements (e.g., “I am satisfied with the appearance of my genitals”) described themselves on a scale of 1 (*does not describe me at all*) to 7 (*describes me very well*). An EFA indicated that the four items of the FGSIS reduced to a single dimension in women and men (see Table S1). Item scores were averaged to form a genital image evaluation score, where higher scores represented more positive evaluations of one’s genital image, ω = 0.87 (95% CI = 0.85, 0.88). Given that the item “I think my genitals smell fine” is excluded in the male equivalent of this scale (Herbenick et al., 2013), we also computed a genital image evaluation score that excluded this item and retested our models using this score. Because our findings did not differ when we used the 4-item and 3-item versions of genital image evaluation, we report results based on the 4-item version of genital image evaluation.

#### Body Size Dissatisfaction

Pulvers et al.’s (2012) 9-figure scale, which features a series of nine sex-specific figure drawings ranging from 1 (*very thin*; approximate body mass index [BMI] ≤ 18) to 9 (*very obese;* approximate BMI ≥ 40) was used to measure body size dissatisfaction. We selected this scale rather than other figure rating scales due to the resemblance of the figures to individuals of multiethnic backgrounds and the greater detail in the drawings. Participants selected the same-sex figure that best represented what they thought they looked like (actual scores) and the figure they desired to look like (ideal scores). In the current study, participants’ actual scores correlated strongly with their BMI (men: *r* = 0.81, *p* < 0.001; women: *r* = 0.81, *p* < 0.001) and self-reported weight in kilograms (men: *r* = 0.79, *p* < 0.001; women: *r* = 0.78, *p* < 0.001). The absolute difference between the ideal score and the actual score was used as an index of body size dissatisfaction, with higher scores representing greater body size dissatisfaction.

#### Satisfaction with Height and Weight

Weight and height satisfaction were measured with single-item measures. Participants rated on a 7-point scale (1 = *extremely dissatisfied* to 7 = *extremely satisfied*) their degree of satisfaction with their current weight and current height.

#### Sexual Health Behaviors

We focused on sexual practices that are associated with a transmission risk of STIs. To capture abstinence from sex, participants reported whether they had ever experienced oral sex (defined as stimulation of genitals using mouth or tongue) and penetrative sex (defined as sexual activity involving penile-anal and/or penile-vaginal penetration). Individuals with sexual experience reported if they had ever had unprotected (condomless) sex, their lifetime number of penetrative sex partners, and if they had ever had a HIV test.

#### Sociodemographic and Other Control Variables

Sociodemographic information included variables for age, gender, race, sexual orientation, partnered sexual experience, and relationship status (see Table 1). Using a single-item measure, participants rated the degree to which they perceived sex as important to their lives on a scale of 1 (*not at all important*) to 7 (*very important*). Individuals with sexual experience reported the frequency and recency of their partnered sexual activity, perceived risk of HIV (“How at risk do you consider yourself of having HIV/AIDS?”), and age of sexual debut (see Table 3 for response options). Religiosity was measured using a single-item measure (“I consider myself a religious person”) rated on a 7-point scale (1 = *definitely not true of me*, 7 = *definitely true of me*). Subjective SES was measured using the McArthur Scale of Subjective Social Status (Adler et al., 2000) with possible scores from 1 to 10, where higher scores indicated higher subjective SES. BMI was computed by dividing one’s self-reported weight in kilograms by the square of height in meters. Participants also reported how often they used recreational drugs every week (see Table 1 for response items).

### Procedure

Data for the current study were collected as part of a larger project on interpersonal experiences and well-being in Malaysian emerging adults that was approved by the Human Research Ethics Committee of the first author’s institution. To qualify for participation, individuals had to be Malaysian by nationality, aged between 18 and 30 years, fluent and able to complete a survey in English, and comfortable with responding to questions about their close relationships and sexual experiences. Data collection was conducted in two phases, with Phase 1 running from June to September 2019 and Phase 2 from May to October 2020, which is within the period of the national lockdown initiated in response to the COVID-19 pandemic in Malaysia.

To recruit the participants, research assistants approached individuals on university grounds, promoted the study on their personal social media accounts, posted on public social media groups, and reached out to public social media accounts. Because of the reliance on convenience sampling, most of the participants were recruited from the state of Selangor and the federal territory of Kuala Lumpur. These are the most populated and most developed areas in Malaysia, and reported the highest rates of new HIV cases in 2022 (Ministry of Health Malaysia, 2022). Individuals who registered for the study were sent a formal invitation to participate via email, in which an explanatory statement and the link to complete the survey online were provided. In the explanatory statement, invitation email, and at the end of the survey, participants were informed that completion of the survey implied consent. Upon completion, participants were debriefed, given 30 MYR (approximately 6.33 USD) for their participation, and asked to sign a document that acknowledged their participation in the study and the receipt of the honorarium. They were also asked to share the study with their contacts.

### Data Analysis

Data were analyzed in SPSS v.28. Little’s missing completely at random (MCAR) test was non-significant for the overall [χ^2^(83) = 97.3, *p* = 0.135] and sexually experienced [χ^2^(89) = 97.2, *p* = 0.260] samples. Approximately 2% of data were missing for our variables of interest and pairwise deletion was used to handle missing data. We first computed the descriptive statistics related to the demographic characteristics and sexual history and practice for the overall sample (*N* = 584) and the subset of participants who reported ever having oral and/or penetrative sex (*N* = 300). Given that the national lockdown in 2020 may have impacted individual’s accessibility to casual sex partners due to travel restrictions or desire for casual sex due to fear of contracting COVID-19, the study phase (0 = *Phase 1*/*2019*, 1 = *Phase 2/2020*) was included as a control variable. Lifetime oral sex, lifetime penetrative sex, and relationship experience were coded as 0 = *no experience* and 1 = *with experience*, and gender was coded as 0 = *men* and 1 = *women*. We then computed *t*-tests to examine whether continuous control variables and body image indices varied by gender and bivariate correlations between variables of interest that were continuous or dichotomous separately for men and women. Because body image indices were similarly associated with both lifetime experience of oral sex and lifetime experience of penetrative sex, we created a new dummy-coded variable that was the combination of the two (coded as 0 = *have not experienced either oral or penetrative sex*, 1 = *have experienced either oral or penetrative sex*) to capture the experience of partnered sex.

To examine associations between body image and dichotomous sexual health outcomes (i.e., experience of partnered sex, condomless sex, and HIV testing), a series of hierarchical logistic regressions were conducted. In these models, age, subjective SES, religiosity, drug use, BMI, perceived importance of sex, relationship experience, and study phase were included in Step 1 as control variables. All body image indices and gender were included in Step 2, followed by the two-way interactions between body image and gender in Step 3 to test for the possible moderating role of gender. Number of lifetime penetrative sex partners, which was originally a count variable, was transformed into an ordinal variable (see Table 3) because the distribution of scores did not follow a Poisson distribution. A hierarchical ordinal regression was computed for this outcome variable. Finally, given that a higher number of sex partners tends to be correlated with higher rates of HIV testing (Ramseyer Winter et al., 2020), we included the number of lifetime penetrative sex partners as an additional control variable in Step 4 of the hierarchical logistic regression if any of the body image indices were found to be significant predictors of HIV testing.

Because the period of emerging adulthood is proposed to last from 18 to 29 years and our dataset contains seven participants who reported ages of 30, sensitivity analyses were run to determine if these participants needed to be excluded from the analyses. Sensitivity analyses revealed no differences in findings. Thus, we reported the results from analyses that included their responses.

## Results

### Preliminary Analyses

Overall, men and women were similar in their self-reported age, subjective SES, drug use, and degree of satisfaction with overall appearance, weight, and body size. Men generally reported higher BMIs, were less religious, perceived sex as more important, were less satisfied with their height, and evaluated their genitals more positively than women (see Table S2).

As shown in Table 2, body image indices were mostly significantly correlated with each other. In general, individuals who were more satisfied with one aspect of their body (e.g., overall appearance) tended to be more satisfied or less dissatisfied with other aspects (e.g., weight, body size) of their body. In contrast, height satisfaction was not associated with body size dissatisfaction among men and women. Among men, there was no significant association between height satisfaction and satisfaction with overall appearance. Men and women with higher BMIs were generally less satisfied with their overall appearance and weight. They also reported a larger discrepancy between their actual and ideal body size (i.e., greater body size dissatisfaction). Women but not men with higher BMIs reported greater satisfaction with their height.

**Table 2:** Bivariate correlations between variables of interest separated by gender (*N* = 584)

Variables	1	2	3	4	5	6	7	8
1. BMI		0.10	0.09	− 0.39***	− 0.39***	0.07	0.42***	− 0.09
2. Ever had oral sex	− 0.02		–	0.03	− 0.03	− 0.01	− 0.00	0.30***
3. Ever had penetrative sex	0.03	–		0.13	− 0.03	− 0.06	− 0.02	0.31***
4. Appearance satisfaction	− 0.21***	0.08	0.14*		0.53***	0.07	− 0.56***	0.42***
5. Weight satisfaction	− 0.41***	− 0.01	0.01	0.51***		0.18**	− 0.51***	0.16*
6. Height satisfaction	0.11*	0.02	− 0.03	0.20***	0.11*		0.05	0.13*
7. Body size dissatisfaction	0.53***	− 0.00	0.03	− 0.50***	− 0.54***	− 0.03		− 0.15*
8. Genital image evaluation	0.08	0.25***	0.24***	0.47***	0.24***	0.20***	− 0.19***	

Coefficients for men are above the diagonal while those for women are below. Ever had oral and penetrative sex were dummy coded as 0 = *no experience* and 1 = *with experience*. Among men, there were four missing values for BMI and two for body size dissatisfaction. Among women, there were nine missing variables for BMI and two for body size dissatisfaction

*p < 0.05, **p < 0.01, ***p < 0.001

Of the 584 participants, 51% reported that they had experienced partnered sex within their lifetime. Specifically, 272 (47%) had experienced oral sex, while 257 (40%) had experienced penetrative (i.e., penile-vaginal or penile-anal) sex. The average age of first non-penetrative sex (defined as any form of partnered sexual activity that did not involve penile-vaginal or penile-anal penetration) was 17.99 years (*SD* = 3.53; range = 4–28) for men and 18.31 years (*SD* = 2.90; range = 6–26) for women. The average age for first penetrative sex was 19.09 years (*SD* = 2.98; range = 8–28) for men and 19.62 years (*SD* = 2.60; range = 14–30) for women.

Among those who had experienced partnered sex (*N* = 300), most reported having partnered sexual activity less than twice a month, mostly within the past three months (Table 3). Despite most participants reporting experience with condomless sex, only a few perceived moderate-to-high risk of HIV and had ever tested for HIV. Of those who reported penetrative sexual experience, most (76%) reported between one and three penetrative sexual partners in their lifetime. Bivariate correlations (Table S2) revealed that men and women who had ever experienced condomless sex and tested for HIV generally reported more penetrative sex partners.

**Table 3:** Sexual practices of participants with partnered sexual experience (*N* = 300)

Variables	N	%
*Frequency of partnered sexual activity*		
Never or almost never	61	20.4
Less than once a month	73	24.4
About once a month	32	10.7
2–3 times per month	62	20.7
Once a week	34	11.4
Multiple times per week	36	12.0
Daily	1	0.3
Missing	1	
*Recency of partnered sexual activity*		
> a year ago	40	13.9
6–12 months ago	27	9.4
3–6 months ago	33	11.5
1–3 months ago	55	19.1
1–4 weeks ago	39	13.5
< a week ago	94	32.6
Missing	12	
*Perceived risk of HIV*		
None at all	207	69.0
A little	60	20.0
A moderate amount	18	6.0
A lot	6	2.0
A great deal	9	3.0
*Ever tested for HIV*		
No	236	78.7
Yes	64	21.3
*Ever had unprotected oral sex*		
No	12	4.4
Yes	260	95.6
Never had oral sex	28	
*Ever had unprotected penetrative sex*		
No	60	23.3
Yes	197	76.7
Never had penetrative sex	43	
*Number of lifetime penetrative sexual partners*		
1	116	45.5
2	48	18.8
3	29	11.4
4	13	5.1
5–6	16	6.3
7–9	9	3.5
10–19	12	4.7
> 20	12	4.7
Missing	2	

### Body Image and the Experience of Partnered Sex

In the hierarchical logistic regression to examine associations between body image indices and one’s experience of partnered sex, no multicollinearity or multivariate outliers were present. Hosmer and Lemeshow tests showed that Steps 1, 2, and 3 were a good fit for the data, *p*s > 0.250. As shown in Table 4, the addition of the interaction terms between gender and body image indices in Step 3 did not improve the model from Step 2, ∆χ^2^(5) = 2.03, *p* = 0.846), indicating that gender did not significantly moderate the associations between body image indices and experience of partnered sex. Genital image evaluation was the only body image index that emerged as a significant predictor in Step 2, *OR* = 1.41, 95% CI [1.18, 1.69], *p* < 0.001 (see Table 4).

**Table 4:** Results from logistic regression testing the associations between negative body image and one’s experience of partnered sex with gender as a moderator

Variables	Step 1	Step 2	Step 3
Age	0.21***	0.20***	0.19***
Subjective SES	− 0.08	− 0.12	− 0.11
Religiosity	− 0.27	− 0.29***	− 0.30***
Drug use	1.04**	1.04**	1.02**
BMI	0.01	0.01	0.01
Importance of sex	0.33***	0.26***	0.27***
Study phase	0.53*	0.53*	0.51*
Relationship experience	2.82***	2.74***	2.76***
Appearance satisfaction		− 0.06	− 0.24
Weight satisfaction		0.03	0.00
Height satisfaction		− 0.09	− 0.05
Body size dissatisfaction		0.02	− 0.21
Genital image evaluation		0.35***	0.45**
Gender		0.06	− 0.44
Appearance satisfaction x gender			0.29
Weight satisfaction x gender			0.04
Height satisfaction x gender			− 0.06
Body size dissatisfaction x gender			0.35
Genital image evaluation x gender			− 0.15
χ^2^ Model	269.90***	287.67***	289.70***
*Cox and Snell R*^*2*^	0.38	0.40	0.40
*Nagelkerke R*^*2*^	0.51	0.53	0.54
∆ χ^2^		17.77**	2.03

Relationship experience was dummy coded as 0 = *no experience* and 1 = *with experience,* gender as 0 = *man* and 1 = *woman,* study phase as 0 = *Phase 1/2019* and 1 = *Phase 2/2020*

*N* = 563 (21 cases were removed due to missing values). Sensitivity analyses revealed that results remain unchanged when the analysis was run without the control variables, with genital image scores that excluded the item “I think my genitals smell fine”, with each body image predictor in their individual models, and separately by study phase

*p < 0.05, **p < 0.01, ***p < 0.001

Results remained unchanged when the body image indices were tested separately in their own models. As shown in Table S4, more positive genital image evaluation was associated with a higher likelihood of experiencing partnered sex, *OR* = 1.35, 95% CI [1.16, 1.58], *p* < 0.001. Other significant predictors included being older, *OR* = 1.20, 95% CI [1.08, 1.34], *p* < 0.001, being less religious, *OR* = 0.75, 95% CI [0.66, 0.85], *p* < 0.001, using recreational drugs more frequently, *OR* = 2.84, 95% CI [1.39, 5.80], *p* = 0.004, viewing sex as highly important, *OR* = 1.31, 95% CI [1.13, 1.52], *p* < 0.001, completing the study in Phase 2, *OR* = 1.72, 95% CI [1.07, 2.76], *p* = 0.026, and having had relationship experience, *OR* = 15.59, 95% CI [8.35, 29.11], *p* < 0.001.

### Body Image and Sexual Health Practices among Participants with Partnered Sexual Experience

#### Number of Lifetime Penetrative Sex Partners

As shown in Table 5, the addition of the interaction terms in Step 3 did not significantly improve the model from Step 2, ∆χ^2^(5) = 4.24, *p* > 0.500), which indicated that gender did not moderate the associations between body image and one’s number of lifetime penetrative sex partners. Results from Step 2 revealed that genital image evaluation was the only significant body image predictor in the model (*p* = 0.007).

**Table 5:** Results of hierarchical ordinal and logistic regressions testing the association between negative body image and sexual health outcomes among participants with partnered sexual experience

Variables	Number of lifetime penetrative sex partners			Condomless			HIV testing			
	Step 1	Step 2	Step 3	Step 1	Step 2	Step 3	Step 1	Step 2	Step 3	Step 4
Age	0.07	0.08	0.08	− 0.02	− 0.02	− 0.03	0.24***	0.24***	0.25***	0.24***
Subjective SES	− 0.03	− 0.03	0.00	− 0.23	− 0.21	− 0.18	− 0.01	− 0.01	0.04	0.04
Religiosity	− 0.12	− 0.13	− 0.16*	0.07	0.05	0.08	− 0.04	− 0.05	− 0.07	− 0.02
Drug use	0.45**	0.51***	0.50***	0.49	0.49	0.51	0.06	0.05	0.01	− 0.12
BMI	0.05*	0.06*	0.06*	− 0.04	− 0.04	− 0.05	− 0.05	− 0.06	− 0.06	− 0.10*
Importance of sex	0.05	0.07	0.08	0.26*	0.27*	0.28*	0.13	0.12	0.13	0.10
Study phase	0.16	0.18	0.20	− 0.09	− 0.09	− 0.13	− 0.16	− 0.17	− 0.18	− 0.34
Relationship experience	0.10	0.60	0.39	1.50*	1.22	1.51*	− 0.49	− 0.52	− 0.77	− 1.06
Appearance satisfaction		0.07	− 0.05		0.23	− 0.16		0.13	− 0.06	0.02
Weight satisfaction		0.00	− 0.04		− 0.13	0.02		− 0.04	0.10	0.13
Height satisfaction		0.01	0.10		− 0.11	− 0.05		− 0.05	0.00	0.02
Body size dissatisfaction		− 0.07	− 0.23		0.06	0.15		0.13	0.19	0.37
Genital image evaluation		− 0.27**	− 0.07		− 0.04	− 0.12		− 0.02	0.38	0.33
Gender		− 0.40	0.64		0.18	− 1.17		− 0.11	3.55	3.64
Appearance satisfaction × Gender			0.22			0.81			0.33	0.08
Weight satisfaction × Gender			0.05			− 0.31			− 0.26	− 0.26
Height satisfaction × Gender			− 0.15			− 0.16			− 0.09	− 0.09
Body size dissatisfaction × Gender			0.26			− 0.11			− 0.13	− 0.26
Genital image evaluation × Gender			− 0.31			0.14			− 0.61*	− 0.44
Number of lifetime penetrative sex partners										0.33***
χ^2^ Model	21.87**	30.98***	35.22*	19.96**	23.42	27.98	16.26*	17.11	24.30	42.12**
*Cox and Snell R*^*2*^	0.09	0.12	0.13	0.08	0.09	0.11	0.06	0.07	0.09	0.15
*Nagelkerke R*^*2*^	0.09	0.12	0.14	0.12	0.14	0.17	0.10	0.10	0.14	0.24
∆− 2 log likelihood		9.11^a^	4.24		3.46	4.57		0.85	7.19^b^	17.82***

Relationship experience was dummy coded as 0 = *no experience* and 1 = *with experience,* gender as 0 = *man* and 1 = *woman,* study phase as 0 = *Phase 1/2019* and 1 = *Phase 2/2020*

Ordinal regression was run to test the model with the number of lifetime penetrative sexual partners as the outcome variable. Logistic regressions were run for the outcome variables condomless sex and HIV testing. *N* = 245 for all analyses

Sensitivity analyses revealed that results remain mostly unchanged when the analysis was run without the control variables, with genital image evaluation scores that excluded the item “I think my genitals smell fine”, with each body image predictor in their individual models, and separately by study phase. The only different finding, which emerged when the analyses were separated by study phase, was a significant appearance satisfaction x gender interaction for the outcome variable condomless sex in the subsample that completed the study in Phase 1 (*N* = 92). However, we refrain from interpreting this significant interaction given the small sample size and its absence in models testing other outcome variables

*p < 0.05, **p < 0.01, ***p < 0.001

^a^The change in − 2 log-likelihood was significant in the model where genital image evaluation was the only body image predictor of interest, χ^2^(2) = 8.51, *p* < 0.025

^b^The change in − 2 log-likelihood was significant in the model where genital image evaluation was the only body image predictor of interest, χ^2^(1) = 5.59, *p* < 0.025

These findings remained stable when the body image indices were tested separately in their own models. As shown in Table S4, individuals with more positive genital evaluation reported fewer lifetime penetrative sex partners, *OR* = 0.78, 95% CI [0.65, 0.94], *p* = 0.007. Other significant predictors included more frequent recreational drug use, *OR* = 1.65, 95% CI [1.23, 2.21], *p* < 0.001, and BMI, *OR* = 1.05, 95% CI [1.00, 1.09], *p* = 0.031.

#### Condomless Sex

None of the body image indices were found to be significantly associated with one’s experience of condomless sex regardless of gender. Findings from Step 1 (Table 5) revealed that individuals who perceived sex as more important, *OR* = 1.30, 95% CI [1.04, 1.62], *p* = 0.020, and who have been in a romantic relationship, *OR* = 4.46, 95% CI [1.20, 16.59], *p* = 0.026, were more likely to have had condomless sex. No other predictors were significant, *p*s > 0.050.

#### HIV Testing

As shown in Table 5, there was a significant interaction between genital image and gender (*p* = 0.044), which was reduced to non-significance after accounting for one’s number of lifetime penetrative sex partners (*p* = 0.155). No other body image indices emerged as significant predictors.

Results remain unchanged in the more parsimonious model where genital image evaluation was tested as the sole body image predictor of HIV testing. The genital image evaluation x gender interaction that was significant in Step 3 (see Table S4), *OR* = 0.54, 95% CI [0.32, 0.92], *p* = 0.023, was reduced to non-significance (*p* = 0.071) after accounting for one’s number of lifetime penetrative sex partners. In this model (Step 4, Table S4), the only significant predictors of HIV testing were a larger number of lifetime penetrative sex partners, *OR* = 1.38, 95% CI [1.18, 1.61], *p* < 0.001, older age, *OR* = 1.29, 95% CI [1.12, 1.49], *p* < 0.001, and having a lower BMI, *OR* = 0.92, 95% CI [0.26, 0.99], *p* = 0.019.

## Discussion

The aim of this study was to examine the role of negative body image indices as predictors of sexual health behaviors in urban Malaysian emerging adults, and whether these associations were moderated by gender. Only partially supporting our first hypothesis (H1) that individuals with lower negative body image (i.e., more satisfied with their bodies) would be more likely to engage in more sexual health behaviors that reduce HIV/STI transmission, our results indicated that the evaluation of one’s genitals had unique associations with ever having had partnered sex and number of lifetime penetrative sex partners. In contrast, however, all other body image indices included in the present study did not emerge as significant predictors. Additionally, lifetime unprotected penetrative sex and HIV testing were not significantly associated with negative body image. Furthermore, and in contrast to our second hypothesis (H2), gender did not significantly moderate associations between body image and sexual health outcomes.

### Body Image and Lifetime Experience of Sex

As sexual abstinence may be one strategy to prevent STI contraction, we first examined whether negative body image was associated with one’s experience of partnered sex. Although the topic of sexual and reproductive health is not openly discussed in Malaysia (Ismail & Abd Hamid, 2016), the prevalence rate of sexual experience in our sample (51%) was similar to what has been found in undergraduate students in the United States (~ 50%; Gillen & Markey, 2014; Schooler et al., 2005). The prevalence rate in the current study was higher than what was found in Durex’s Sexual Health and Intimate Wellness Survey that was conducted with 1089 Malaysians aged 18–30 years from April to May 2022 (Hassandarvish, 2022; 35%) and an earlier survey with 1327 students from public universities in Selangor, who were predominantly Muslim (98%) and single (71%; Sham et al., 2020). The higher prevalence in our sample may be due to our reliance on convenience sampling and our inclusion criteria, namely being comfortable with completing a survey in the English language and responding to questions about close relationships and sexual experiences. This may have discouraged potential respondents with more conservative values about sex from participating.

Men and women with more positive genital image evaluation were more likely to have engaged in partnered sex, even after accounting for age, drug use, religiosity, perceived importance of sex, and relationship experience. A possible explanation is that individuals who evaluate their genitals more positively feel more comfortable exposing their genitals during sex and are therefore more confident when seeking out sexual experiences (Reinholtz & Muehlenhard, 1995). This is in line with previous studies showing that more positive genital self-image is associated with higher sexual desire (Berman et al., 2003; Zielinski et al., 2012) and greater confidence as a sexual partner (Amos & McCabe, 2016). This finding is also consistent with the argument based on objectification theory (Fredrickson & Roberts, 1997) that feeling more positive about one’s body reduces one’s fear of negative appearance-based evaluation and avoidance of bodily exposure. However, the causal direction of this association is unclear. It is equally possible that emerging adults who are sexually active hold more positive attitudes and feelings toward their genitals because they make the (corrective) experience that their romantic or sexual partner(s) find their bodies sexually appealing.

Beyond genital image evaluation, no other body image indices (overall appearance satisfaction, body size dissatisfaction, satisfaction with height and weight) were associated with lifetime partnered sex. Similarly, some studies have found body satisfaction and lifetime sexual experience to be unrelated (e.g., Wiederman & Hurst, 1998), while others have found a positive association (e.g., Merianos et al., 2013). These mixed findings may be in part due to the variability in body image measures used. In the current study, findings from bivariate analyses suggest that generally, women with penetrative sex experience tended to be more satisfied with their overall appearance than women without penetrative sex experience. However, this association disappeared once the effects of other body image indices and control variables were considered. Our finding of the superior role of genital image evaluation in predicting lifetime sex relative to other body image indices is consistent with past findings on the role of genital image in female sexual health behaviors (DeMaria et al., 2012; Pakpour et al., 2014) and breast size dissatisfaction in breast health behaviors (Swami & Furnham, 2018).

Apart from their direct relevance to sexual activity, genitals are body parts that are (typically) less publicly exposed and thus less vulnerable to evaluation by others as compared to one’s overall appearance, weight, height, and body size. However, this also translates into fewer opportunities for one to obtain feedback on their potentially biased evaluations of their genitals. Up to the first partnered sexual experience, genital image (dis)satisfaction is predominantly influenced by an individual’s evaluation based on mainstream representations of the genital appearance in media images or sociocultural beliefs, which may be especially unsettling for emerging adults with a lack of knowledge of the basic anatomical features and diversity of the genitals (Fernando & Sharp, 2020). Given the sexual conservativeness observed in Malaysia, it is perhaps unsurprising that genital image is more strongly associated with one’s feelings, thoughts, and behaviors related to sex. Although not measured in the current study, the different mechanisms underlying one’s development of genital image as compared to other body image indices may also explain its unique association with one’s experience of partnered sex.

### Body Image and Number of Lifetime Penetrative Sex Partners

Among participants who have engaged in partnered sex, only genital image evaluation emerged as a significant predictor of the number of lifetime sex partners. Similar to our finding on the association between body image and experience of partnered sex, no other body image indices were associated with the number of sex partners, which supports past findings on the superior contribution of region-specific body image over global body image indices (DeMaria et al., 2011, 2012; Pakpour et al., 2014; Swami & Furnham, 2018). Second, our finding that genital image evaluation was associated with a lower likelihood of abstaining from sex but also with having fewer sexual partners suggests that body image can contribute to various forms of preventive sexual health practices in different ways. For instance, Schick et al. (2010) found that women who were less dissatisfied with the appearance of their genitals reported less self-consciousness during sex, which was associated with heightened self-perceived value as a sexual being (i.e., higher sexual esteem). This higher sexual esteem, in turn, predicted greater sexual satisfaction and motivation to avoid risky sexual behaviors. Similar to Schick et al. (2010), our findings suggest that even though more positive genital image evaluation may increase one’s engagement in and enjoyment of sexual activity (Reinholtz & Muehlenhard, 1995), it may also limit one’s sexual network as a result of heightened sexual esteem. Notably, our findings indicate that these associations also apply to male emerging adults.

### Body Image, Condomless Sex, and HIV Testing

Only about 20% of our sexually experienced participants had ever tested for HIV despite over 75% of them reporting having had condomless sex. However, this may be due to the relatively low number of lifetime penetrative sex partners reported by our participants: 45% reported only one partner while 30% reported two to three partners. The HIV testing rate found in the current study is comparable to the rates found in general adult populations (18 years and above) in Malaysia (20%; Lemin et al., 2020; Wong, 2012) and in undergraduate students in the United States (30%; Gillen & Markey, 2014).

Overall, we found no evidence for the role of negative body image in condomless sex and HIV testing. This is consistent with findings from a study that was conducted with college students in the United States in their emerging adulthood (Gillen & Markey, 2014), but inconsistent with Ramseyer Winter et al. (2020) who found that feeling less negative about one’s body was associated with HIV or STI testing among men who engage in casual sex. These findings suggest that null associations may be a reflection of the lower sexual knowledge and experience in the current sample of Malaysian emerging adults, which was similar to Gillen and Markey (2014) in the prevalence of lifetime sex and HIV testing. Body image may be a less important predictor of lifetime unprotected sex when individuals do not have sufficient knowledge about the health risks that come with condomless sex that go beyond unwanted pregnancies, thus resulting in a potential ceiling effect. Indeed, our findings are consistent with previous findings that highlight the stronger contribution of behavioral predictors in predicting sexual health practices as compared to attitudinal predictors such as body image (DeMaria et al., 2011). For instance, a higher number of sexual partners was the most reliable predictor of HIV testing found in our study.

Overall, we found that emerging adults with more positive genital image evaluations were more likely to have had partnered sex. Among men and women who had sexual experience, more positive evaluation of genitals was associated with fewer sexual partners. To the best of our knowledge, the current study was the first to extend the examination of the link between genital image and sexual health practices to men and a Southeast Asian context. Condomless sex and HIV testing were predicted by sexual and relationship experience and not body image. Overall, the associations between negative body image indices and sexual health outcomes were consistent across genders. We speculate that this may be due to the similarities in sexual health practices among men and women in our sample, which is likely more prevalent among populations that hold more conservative beliefs about sex. For instance, Sohn and Chun (2007) found among Korean emerging adults that, even though men reported more sexual experience than women, there were no gender differences in condom use. We ran post hoc analyses, which controlled for age and sexual orientation, and found that while men were more likely to report sexual experience than women, they did not differ in lifetime HIV testing, condomless sex, and number of penetrative sex partners. To shed light on these speculations, future work could examine the role of conservatism, sexual knowledge and experience, and sex guilt or shame as potential suppressors of body image or gender effects in sexual health practices. It would also be worthwhile to examine whether the less pronounced contribution of global body image indices is underlined by the more sexually conservative context of the current sample.

### Limitations

Our findings should be interpreted in consideration of the limitations of our study. First, we relied on a cross-sectional design, which does not allow us to make conclusions about the causal effects of body image on sexual health practices. Despite our proposition based on previous theoretical and empirical evidence on the causal role of body image, it is also possible that our findings reflect the role of sexual health practices leading to body image outcomes. To address this limitation, future work should consider using longitudinal study designs, which employ repeated measurements over time, and include measures of relevant constructs to directly test the applicability of our theoretical framework (i.e., objectification theory). Second, we only measured whether participants had ever engaged in condomless sex or HIV testing in their lifetime rather than their recent practices. We also did not examine whether the HIV testing was part of routine health screening and whether participants had screened for other STIs.

Our participants were largely Chinese Malaysian, which is not representative of the racial breakdown in the Malaysian population. While one could argue that the role of body image may be less pronounced among more religious communities (e.g., Penhollow et al., 2007) in Malaysia, such as Malays who are (by national law) Muslims, there is evidence from a study conducted with sexually active adolescents in Singapore—which was previously a part of Malaysia and thus shares many historical and cultural influences—that found riskier sexual practices among Malay than Chinese participants (Ng & Wong, 2019). Moreover, our findings were mostly consistent with theoretical propositions and past empirical findings and were derived from analyses that included the degree of religiosity among other variables as control variables. Finally, we used measures of body image that were originally developed for use with Western populations. There may be value in conducting more emic research in the future, which may help determine whether there are other body image variables that are more strongly associated with sexual health practices in Malaysia.

### Conclusion

Taken together, our findings suggest that the way Malaysian emerging adults evaluate their bodies, and especially their genitals, is associated with their sexual health practices, which has implications for intervention programs to increase sexual health practices that promote well-being and minimize potential harm to overall health. First, such programs should incorporate elements of promoting more positive genital image evaluation. For instance, interventions that include challenging body ideals (Williamson & Karazsia, 2018) and genital appearance ideals (e.g., Fernando & Sharp, 2020) could be helpful. More importantly, any intervention to promote sexual health needs to be combined with education related to sexual attitudes, knowledge, and practices that are culturally inclusive and promote safer sex practices. Such holistic sex education programs need to be made readily available, especially among emerging adults who engage in unprotected sex and yet do not screen for HIV or other STIs.

### Supplementary Information

Below is the link to the electronic supplementary material.

Supplementary file1 (DOCX 33 KB)

## Data Availability

The data that support the findings of this study are available from the corresponding author upon reasonable request.
