# Supplementary material for: Associations Between Negative Body Image and Sexual Health Practices in Emerging Adults from Malaysia
Source: Arch Sex Behav. 2024 Feb 6;53(4):1473–86. doi: 10.1007/s10508-024-02810-y (PMC10954872; doi:10.1007/s10508-024-02810-y)
Supplement: Supplementary file 1 — Supplementary file1 (DOCX 33 KB) [file 10508_2024_2810_MOESM1_ESM.docx]

**Table S1**. Summary of results from exploratory factor analyses for body measures using principal axis factoring with varimax rotation.

| Variable | n | Bartlett's test of sphericity, χ2 | KMO | Communalities | Number of factors with λ > 1.0 | % of variance explained |
| --- | --- | --- | --- | --- | --- | --- |
| Appearance satisfaction |  |  |  |  |  |  |
| Men | 230 | 837.72^***^ | 0.89 | .39-.69 | 1 | 55.11 |
| Women | 354 | 1089.01^***^ | 0.86 | .36-.69 | 1 | 49.15 |
| Genital image |  |  |  |  |  |  |
| Men | 230 | 474.31^***^ | 0.82 | .38-.76 | 1 | 63.68 |
| Women | 354 | 622.94^***^ | 0.82 | .49-.73 | 1 | 60.33 |

*Notes*. ^***^ *p* < .001.

**Table S2**. Results from independent t-tests examining gender differences in continuous variables of interest in the overall sample.

|  | Men | | |  | Women | | |  |  |
| --- | --- | --- | --- | --- | --- | --- | --- | --- | --- |
| Variable | *M* | *SD* | *N* |  | *M* | *SD* | *N* | *t* | *d* |
| Age | 22.12 | 2.57 | 230 |  | 22.10 | 2.34 | 352 | 0.12 | .01 |
| Body mass index | 23.81 | 5.67 | 226 |  | 21.73 | 4.37 | 345 | 4.89^***^ | .42 |
| Religiosity | 3.27 | 1.83 | 230 |  | 3.65 | 1.75 | 354 | -2.56^*^ | .22 |
| Subjective socioeconomic status | 6.22 | 1.52 | 229 |  | 6.38 | 1.39 | 354 | -1.28 | .11 |
| Importance of sex | 4.81 | 1.50 | 230 |  | 3.76 | 1.70 | 354 | 7.82^***^ | .65 |
| Drug use | 1.18 | 0.64 | 230 |  | 1.12 | 0.52 | 354 | 1.32 | .12 |
| Appearance satisfaction | 3.07 | 0.93 | 230 |  | 3.18 | 0.86 | 354 | -1.45 | .12 |
| Weight satisfaction | 3.72 | 1.82 | 230 |  | 3.66 | 1.78 | 354 | 0.44 | .04 |
| Height satisfaction | 4.16 | 1.75 | 230 |  | 4.52 | 1.70 | 354 | -2.46^*^ | .21 |
| Body size dissatisfaction | 1.21 | 0.97 | 228 |  | 1.23 | 0.94 | 352 | -0.15 | .01 |
| Genital image evaluation | 4.88 | 1.42 | 230 |  | 4.33 | 1.56 | 354 | 4.33^***^ | .37 |

*Notes*. ^*^ *p* < .05, ^***^ *p* < .001.

**Table S3.** Bivariate correlations between demographic variables, body image indicators, and sexual health outcomes separated by gender among participants with partnered sexual experience.

| Variables | 1 | 2 | 3 | 4 | 5 | 6 | 7 | 8 | Men  *M (SD)* |
| --- | --- | --- | --- | --- | --- | --- | --- | --- | --- |
| 1. Appearance satisfaction |  | .52^***^ | .00 | -.61^***^ | 0.32^***^ | -.10 | .01 | .09 | 3.14 (0.89) |
| 2. Weight satisfaction | .52^***^ |  | .08 | -.58^***^ | .09 | -.13 | -.03 | .08 | 3.64 (1.83) |
| 3. Height satisfaction | .21^**^ | .09 |  | .14 | .12 | .02 | -.05 | .02 | 4.13 (1.76) |
| 4. Body size dissatisfaction | -.57^***^ | -.59^***^ | .02 |  | -.11 | .07 | .01 | -.01 | 1.19 (0.96) |
| 5. Genital image evaluation | .40^***^ | .18^*^ | .25^***^ | -.20^**^ |  | .07 | -.02 | .16 | 5.29 (1.27) |
| 6. Lifetime number of penetrative sex partners | .01 | -.12 | -.06 | .07 | -.14 |  | .22^*^ | .29^**^ | 2.87 (2.28) |
| 7. Condomless sex | .06 | -.12 | -.09 | -.00 | -.00 | .19^*^ |  | - | - |
| 8. HIV testing | .03 | -.01 | -.11 | .01 | -.07 | .28^***^ | - |  | - |
| Women  *M (SD)* | 3.30 (0.87) | 3.72 (1.80) | 4.52 (1.71) | 1.22 (0.93) | 4.76 (1.45) | 2.40 (1.93) | - | - |  |

*Notes*. ^*^*p* < .05, ^**^*p* < .01, ^***^*p* < .001.

Coefficients for men are above the diagonal while those for women are below.

**Table S4**. Results from regressions testing the associations between genital image and sexual health outcomes.

|  | Partnered sexual experience |  |  | Number of lifetime penetrative sex partners |  |  | HIV testing |  |  |  |
| --- | --- | --- | --- | --- | --- | --- | --- | --- | --- | --- |
| Variables | Step 1 | Step 2 |  | Step 1 | Step 2 |  | Step 1 | Step 2 | Step 3 | Step 4 |
| Age | 0.20^***^ | 0.18^***^ |  | 0.07 | 0.08 |  | 0.26^***^ | 0.25^***^ | 0.26^***^ | 0.26^***^ |
| Subjective socioeconomic status | -0.07 | -0.11 |  | -0.03 | -0.02 |  | -0.02 | -0.01 | 0.02 | 0.02 |
| Religiosity | -0.27^***^ | -0.29^***^ |  | -0.12 | -0.13 |  | -0.03 | -0.03 | -0.07 | -0.02 |
| Drug use | 1.03^**^ | 1.04^**^ |  | 0.45^**^ | 0.50^***^ |  | 0.06 | 0.06 | 0.01 | -0.13 |
| Body mass index | 0.01 | 0.01 |  | 0.05^*^ | 0.05 |  | -0.05 | -0.05 | -0.05 | -0.09^*^ |
| Importance of sex | 0.33^***^ | 0.27^***^ |  | 0.05 | 0.07 |  | 0.14 | 0.14 | 0.15 | 0.12 |
| Study phase | 0.53^*^ | 0.54^*^ |  | 0.16 | 0.18 |  | -0.16 | -0.16 | -0.18 | -0.33 |
| Relationship experience | 2.83^***^ | 2.75^***^ |  | 0.10 | 0.54 |  | -0.51 | -0.45 | -0.84 | -1.13 |
| Genital image evaluation |  | 0.30^***^ |  |  | -0.25^**^ |  |  | -0.04 | 0.36 | 0.33 |
| Gender |  | -0.02 |  |  | -0.40 |  |  | 0.07 | 3.19^*^ | 2.65 |
| Genital image x Gender |  |  |  |  |  |  |  |  | -0.62^*^ | -0.50 |
| Number of lifetime penetrative sex partners |  |  |  |  |  |  |  |  |  | 0.32^***^ |
|  |  |  |  |  |  |  |  |  |  |  |
| χ^2^ Model | 270.35^***^ | 285.88^***^ |  | 21.87^**^ | 30.39^***^ |  | 18.70^*^ | 18.82^*^ | 24.42^*^ | 41.58^***^ |
| *Cox and Snell R^2^* | .38 | .40 |  | .09 | .12 |  | .07 | .07 | .09 | .16 |
| *Nagelkerke R^2^* | .51 | .53 |  | .09 | .12 |  | .11 | .11 | .14 | .23 |
| ∆ -2 log likelihood |  | 15.54^***^ |  |  | 8.52^**^ |  |  | 0.12 | 5.59^*^ | 17.16^***^ |

*Notes*. ^*^*p* < .05, ^**^*p* < .01, ^***^*p* < .001.

Relationship experience was dummy coded as 0 = *no experience* and 1 = *with experience,* gender as 0 = *man* and 1 = *woman,* study phase as 0 = *Phase 1/2019* and 1 = *Phase 2/2020*.

Ordinal regression was run to test the model with the number of lifetime penetrative sex partners as the outcome variable. Logistic regressions were run for the outcome variables partnered sexual experience and HIV testing.
